# Venous 3D Phase Contrast Magnetic Resonance Angiography Increases Diagnostic Certainty in Children with Ventriculoperitoneal Shunt and Suspected Shunt Failure

**DOI:** 10.1007/s00062-023-01310-1

**Published:** 2023-07-03

**Authors:** M. Huhndorf, S. Peters, J. Cordt, N. G. Margraf, M. Salehi Ravesh, O. Jansen, M. Synowitz, G. Cohrs

**Affiliations:** 1 Department of Radiology and Neuroradiology, University Hospital Schleswig-Holstein, Campus Kiel, Kiel, Germany; 2 Department of Neurology, University Hospital Schleswig-Holstein, Campus Kiel, Kiel, Germany; 3 Department of Neurosurgery, University Hospital Schleswig-Holstein, Campus Kiel, Kiel, Germany; 4 Pediatric Neurosurgery, Campus Virchow Klinikum, Charité Universitätsmedizin Berlin, Berlin, Germany

**Keywords:** Hydrocephalus, Intracranial pressure, CSF dynamics, Venous sinus, Ventriculomegaly

## Abstract

**Background:**

Clinical symptoms in children with suspected malfunction of ventriculoperitoneal shunt may not be specific and difficult to interpret. The presence or absence of ventricular enlargement on magnetic resonance imaging (MRI) does not reliably predict raised intracranial pressure (ICP) in these patients. Therefore, the aim was to investigate the diagnostic utility of 3D venous phase-contrast MR angiography (vPCA) in these patients.

**Materials:**

The MR studies of two groups of patients at two different examination dates were retrospectively analyzed; one group without clinical symptoms on both examinations and one with symptoms of shunt dysfunction on one examination receiving surgery. Both MRI examinations had to have been performed including axial T_2_ weighted (T_2_-w) images and 3D vPCA. Two (neuro)radiologists evaluated T_2_-w images alone and in combination with 3D vPCA in terms of suspected elevated ICP. Interrater reliability, sensitivity and specificity were assessed.

**Results:**

Compression of venous sinuses was seen significantly more often in patients with shunt failure (*p* = 0.00003). Consequently, evaluation of 3D vPCA and T_2_-w images increases sensitivity to 0.92/1.0 compared to T_2_-w images alone with 0.69/0.77, the interrater agreement for the diagnosis of shunt failure rises from κ = 0.71 to κ = 0.837. Concerning imaging markers, three groups could be identified in children with shunt failure.

**Conclusion:**

In accordance with the literature, the results show that ventricular morphology alone is an unreliable marker for elevated ICP in children with shunt malfunction. The findings confirmed 3D vPCA as a valuable supplemental diagnostic tool improving diagnostic certainty for children with unchanged ventricular size in cases of shunt failure.

## Introduction

With an incidence of 1–2/1000 births, hydrocephalus is one of the most common conditions affecting the central nervous system in the pediatric population [1]. Ventriculoperitoneal shunting is still the mainstay of treatment. Nevertheless, this kind of hydrocephalus treatment is prone to malfunction and these patients present regularly at hospitals with a variety of symptoms that may be related to shunt failure (SF) or dysfunction [2]. In spite of technical advances concerning shunt design and patient management, dysfunction rates remain high, with about 40% of pediatric and 29% of adult patients undergoing surgery for SF in the first year after shunt insertion. Indeed, at least 1 surgical shunt revision procedure is performed in 45–81% of shunt patients [3–6]. The importance of prompt diagnosis and operative treatment of blocked ventriculoperitoneal shunts cannot be overemphasized as delayed treatment might result in major neurological sequelae or death [7]. So far, there is no gold standard for the preoperative diagnosis of SF [8, 9]. Diagnosis is primarily based on clinical symptoms, such as headache, nausea, vomiting or altered consciousness, and the assessment of ventricular width by using imaging techniques; however, assessment of clinical symptoms, which may vary enormously is often challenging, especially in younger children. Consequently, adequate imaging is essential to evaluate potential shunt dysfunction.

Up to now, computed tomography (CT) examinations have been used to identify patients at risk for SF [9–11]; however, this technique carries the burden of ionizing radiation and cohort studies have shown an increased risk for neoplastic disorders [12, 13]. Emerging techniques, such as quick brain MRI are used in children with shunt dysfunction or traumatic brain injury in order to spare patients from radiation exposure [14, 15]. Still, ventricular size alone does not necessarily predict or rule out SF on CT or MRI [16, 17]. In about 10% of patients with SF ventricular size is normal/unaltered on imaging. Consequently, ventricular size should not be taken as the only definitive diagnostic sign when evaluating patients with SF. A diagnostic concept more adapted to the dynamic changes in intracranial pressure (ICP) might be helpful. In the presence of idiopathic intracranial hypertension, the narrowing of dural sinuses has been described and used as a diagnostic sign as also seen in patients with slit-ventricle syndrome [18–20]. Narrowing of dural sinuses leads to changes in blood flow which can be visualized by 3D venous phase contrast angiography (vPCA). Thus, MRI imaging can combine morphologic and functional imaging.

In order to evaluate vPCA as an additional tool in the diagnostic work-up of potential shunt dysfunction, we investigated MRI examinations of a cohort of pediatric patients with shunted hydrocephalus with and without elevated ICP. In cases with elevated ICP patients underwent shunt revision with clinical improvement after surgery. We additionally hypothesize from yearlong experience that shunted children can be divided into three groups in the case of SF with respect to widening of the ventricles and/or sinus compression with (1) widening of the ventricles without venous sinus compression, (2) widening of the ventricles with venous sinus compression and (3) venous sinus compression without widening of the ventricles.

## Material and Methods

### Data Acquisition

We retrospectively identified children with ventriculoperitoneal shunt who underwent at least two MR examinations from May 2010 to May 2022 with T_2_ weighted (T_2_-w) image in axial plane and 3D vPCA. These children were divided into two groups. Group 1 represents children who underwent operative shunt revision due to SF and in whom MRI had been performed both in the healthy clinical state as well as in the state of suspected shunt dysfunction according to characteristic clinical symptoms. Group 2 represents children who underwent MR examination regarding to routine controls in the healthy condition. Patients included with a long time interval were at least 4 years old to prevent that image impression was affected by fusion of the fontanelles. Shunt dysfunction was defined by improvement of clinical symptoms after revision surgery.

### MR Imaging

MR imaging was performed on a 1.5 T or 3 T whole-body MRI system (1.5 and 3 T, Achieva and Achieva TX-Series, Philips Healthcare, Best, The Netherlands and 1.5 and 3 T, Magnetom Aera and Magnetom Vida, Siemens Healthcare, Erlangen, Germany). Due to the use of different MRI systems and the long period of retrospective data acquisition of 12 years, imaging parameters varied somewhat concerning repetition time (TR)/echo time (TE) of axial 2D T_2_-w images. T_2_-w images were acquired using spin echo sequences with a duration between 3:24 and 4:20 min. TR and TE ranged from 4049–6900 and 100–127 ms, respectively. Slice thickness was 2 or 3 mm, except for one examination with 4 mm, while in-plane resolution ranged from 0.42 to 0.65 mm^2^. 3D vPCA was performed using a T_1_-weighted gradient echo sequence with velocity encoding (VENC) of 15 cm/s in all three spatial directions/planes. Only one examination was performed with VENC of 20 cm/s in two directions. The total acquisition duration ranged from 6:20–8:00 min. Slice thickness was between 0.6 mm^2^ and 1.6 mm^2^ while in-plane resolution ranged from 0.43–0.9 mm^2^. Echo time was 7–9 ms, while repetition time was between 17 and 66 ms.

### Image Analysis

Two board certified radiologists with 5 (rater 1; J.C.) and 11 (rater 2; S.P.) years of experience in neuroradiology evaluated all MR imaging datasets blinded for clinical symptoms and examination dates in separate sessions. In a first analysis only T_2_-w images in axial plane at both examination dates were analyzed and rated according to image quality and ventricular width. On that basis the raters evaluated for each examination if elevated ICP was suspected. The raters were able to compare T_2_-w images of both examination dates.

A second analysis in a time interval of at least 4 weeks was performed on all MR datasets comprising T_2_-w images in axial plane as well as 3D vPCA at both examination dates. Radiologists now had to evaluate both T_2_-w images and 3D vPCA according to image quality, assess ventricular width as well as evaluate if superior sagittal sinus (SSS) or left/right transverse sinus (LTS/RTS) is compressed. Again, the raters had to conclude if they would suspect elevated ICP, being able to compare T_2_-w images and 3D vPCA of both examination dates.

Evaluation scales were as follows:

Image quality of T_2_-w images in axial plane:

- Grade 1: good/excellent image quality with no/minimal artefacts and confident evaluation of brain tissue.
- Grade 2: moderate image quality with sufficient delineation of ventricles and moderate artefacts; limited evaluation of brain tissue.
- Grade 3: poor image quality with significant artefacts, no reliable delineation of ventricles, evaluation of brain tissue impossible.

Image quality of 3D vPCA:

- Grade 1: good/excellent image quality with no/minimal artefacts and confident evaluation of venous sinuses.
- Grade 2: moderate image quality with moderate artefacts and sufficient evaluation of venous sinuses.
- Grade 3: poor image quality with significant artefacts, no reliable evaluation of venous sinuses.

Ventricular size:

- Grade 1 = small
- Grade 2 = normal
- Grade 3 = large

Sinuscompression:

- Yes/No

Elevated ICP/Shunt dysfunction suspected:

- Yes/No

Because within the categories of small, normal and wide ventricles, there can still be changes in ventricular size, a third board certified neuroradiologist with 10 years of experience (MH) analyzed all datasets of children with SF, if obvious changes in ventricular width were visible, to prove our hypothesis that in cases of SF children can be divided into three groups with (1) widening of the ventricles without venous sinus compression, (2) widening of the ventricles with venous sinus compression and (3) venous sinus compression without widening of the ventricles. Changes of ventricular width were evaluated on the level of the frontal horns, the temporal horns, the third ventricle and the fourth ventricle. These results were then combined with results of rater 1 and rater 2 on venous sinus compression (yes/no) to assign to one of the three groups.

### Data Analysis

Diagnostic accuracy statistics including sensitivity and specificity were calculated for the diagnosis of elevated ICP for both analysis of T_2_-w images alone and as well as T_2_-w image and 3D vPCA. Inter-rater reliability for ventricular width, compression of SSS, RTS and LTS as well as elevated ICP was quantified by using κ statistics. For calculation of inter-rater reliability for ventricular width quadratic weighted kappa was used [21]. A κ value of 0.00–0.20 indicates slight agreement, 0.21–0.40 fair agreement, 0.41–0.60 moderate agreement, 0.61–0.8 substantial agreement and 0.81–1.00 indicates almost perfect agreement [22]. Fisherʼs exact test was used to test the difference of venous sinus stenosis between the two groups for significance with alpha level ≤ 0.05.

Ethical review was obtained for this study from the institution’s review board (D 404/20).

## Results

### Patients

In total, 20 patients (13 male) with 2 MRI examinations each were included in the study, of which 13 patients presented with clinical symptoms of potential shunt dysfunction at 1 examination date and therefore underwent operative shunt revision. They all showed marked improvement of clinical symptoms after surgery. Patients’ age ranged from 1 to 16 years with a median of 10 years for the first evaluated MR examination and from 1 to 17 years with a median of 10 years for the second evaluated MR examination. Demographic and clinical information are summarized in Table 1. The time interval between both MR examinations ranged from 4 days to 3.5 years.

**Table 1:** Demographic and clinical patient information

Number of patients	20
Number of examinations	40
Number of examinations with shunt dysfunction	13
Female/male	7/13
Age (years) at MRI 1	1–16 (median = 10)
Age (years) at MRI 2	1–17 (median = 10)
*Cause of hydrocephalus*	
PHH after IVH/ICH	8
Myelomeningocele	4
Malresorption	1
Postinfection	2
Pseudotumor cerebri	1
Crouzon syndrome	1
Aqueduct stenosis/occlusion	3

*PHH* posthemorrhagic hydrocephalus, *IVH* intraventricular hemorrhage, *ICH* intracerebral hemorrhage, *MRI* magnetic resonance imaging

### Image Analysis

Image quality of T_2_-w images was rated as good/excellent in 100% by rater 1 and 87.5% by rater 2. Rater 2 described 12.5% as moderate image quality. Image quality of 3D vPCA was rated as good/excellent in 80% by rater 1 and 97.5% by rater 2. Moderate image quality was described in 20% by rater 1 and 2.5% by rater 2. In only one of the cases were the MR examinations made on different MRI systems with the same magnetic field strength, but different vendors.

Ventricular size was evaluated as small/normal/large in 18/10/12 of 40 examinations by rater 1 and in in 21/7/12 of 40 examinations by rater 2. Inter-rater reliability for ventricular width on T_2_-w images was κ = 0.85.

Sinus compression was observed in 14/40 and 13/40 examinations by rater 1 and rater 2, respectively. Both raters identified significantly more sinus compressions in MR examinations with symptoms of SF with 11/13 and 10/13 examinations for rater 1 and rater 2, respectively, while both raters identified sinus compression in only 3/27 routine MR examinations without symptoms of SF (*p* = 0.00003). Inter-rater reliability for compression of the venous sinus showed almost perfect agreement for SSS and RTS with κ = 0.88 and κ = 0.8, respectively and substantial agreement with κ = 0.69 for the LTS.

Inter-rater reliability for suspected elevated ICP showed substantial agreement with κ = 0.71 for evaluation of T_2_-w images alone and almost perfect agreement with κ = 0.837 for the combination of T_2_-w images and 3D vPCA.

Sensitivity and specificity for detection of SF by evaluating T_2_-w images alone was 0.69 and 0.82 for rater 1 and 0.77 and 0.96 for rater 2, respectively. By evaluating T_2_-w images and 3D vPCA sensitivity and specificity were 0.92 and 0.93 for rater 1 and 1.0 and 0.93 for rater 2, respectively. All values are summarized in Table 2.

**Table 2:** Predictive values of MRI examinations for rater 1 and rater 2 as well as inter-rater reliability for evaluation of T_2_-weighted images alone and in combination with 3D vPCA concerning shunt dysfunction

	T_2_-w		T_2_-w + 3D vPCA	
	Rater 1	Rater 2	Rater 1	Rater 2
True positive	9	10	12	13
True negative	22	26	25	25
False positive	5	1	2	2
False negative	4	3	1	0
Sensitivity	0.69	0.77	0.92	1.0
Specificity	0.82	0.96	0.93	0.93
Inter-rater reliability	0.71		0.84	

*T2-w* T2-weighted, *vPCA* venous phase contrast MR angiography

**Fig. 1:**
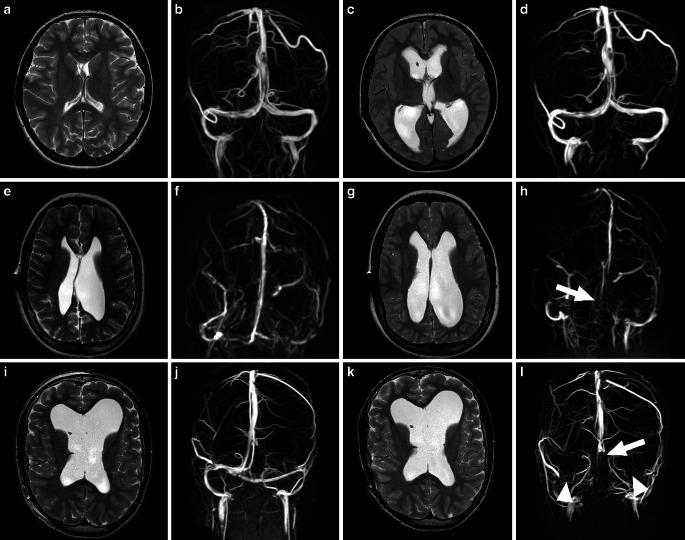
Axial T2 weighted images and 3D venous phase contrast MR angiography in a coronal view of 3 patients in the healthy clinical state (the two *left images* of a row) and with elevated intracranial pressure (ICP) due to shunt failure (the two *right images* of a row). **a–d** Show a patient only presenting with distinct widening of the ventricles without involvement of the venous sinuses (group 1). **e–h** Show a case where elevated ICP is associated with widening of the ventricles as well as compression of the superior sagittal sinus (*white arrow*) (group 2) and **i–l** show a patient with unchanged ventricular size but distinct compression of the superior sagittal sinus (*white arrow*) as well as the left and right transverse sinus (*white arrowheads*) (group 3)

The reading of all examinations of children with shunt failure (SF) by rater 3 revealed a compression of venous sinus in 85% (11/13) of the cases with SF, of whom 36.4% (4/11) showed no increasing ventricle width. The children could be assigned to the 3 previously defined groups with 2/13 (15.4%) children assigned to group 1, 7/13 (54%) children assigned to group 2 and 4/13 (31%) children assigned to group 3. Examples for each group are visualized in Fig. 1.

## Discussion

The aim of this study was to identify an additional imaging tool to gain further diagnostic certainty in shunt dysfunction associated with underdrainage in children. In agreement with the literature, the results show that ventricular enlargement alone is not a sensitive marker for potentially raised ICP in shunted children [9–11, 23, 24]. The results of this study show that an additional evaluation of the venous sinuses using 3D vPCA substantially increases diagnostic sensitivity in children with suspected shunt dysfunction compared to evaluation of T_2_-w images alone. Both raters observed significantly more compressed venous sinuses in examinations with SF than in routine scans. Sensitivities of 0.92 and 1.0 for both raters, respectively, yielded that 3D vPCA therefore represents a useful additional tool in clinical routine. In our study sensitivities of 0.69 and 0.77 just evaluating T_2_-w images are quite high compared to other studies evaluating ventricle size with sensitivities between 0.5 and 0.65 using CT or quick brain MRI [24–26]. An explanation might be that the raters are aware of venous sinus changes due to elevated ICP and therefore unconsciously included hints concerning changes in the venous system visible on T_2_-w images. Consequently, increase in diagnostic sensitivity might be even greater in hospitals where evaluation of venous structures in suspected elevated ICP is not routinely performed.

Inter-rater reliability for evaluation of compression of SSS and LTR/RTS with κ values indicating substantial and almost perfect agreement shows that this does not lead to different assessments and even increases inter-rater reliability for overall evaluation regarding suspected shunt dysfunction. 3D vPCA is easy to perform but takes slightly longer than acquiring T_2_-w images in the axial plane alone, which could also be performed as fast or single-shot sequences. As ventricular width does not detect shunt dysfunction in about 10% of shunted children [17], 3D vPCA is needed to confirm the diagnosis of raised ICP. Furthermore, image interpretation does not require longstanding experience. Only considering changes in ventricular width would have led to 10% and 7.5% false negative diagnoses in this study for rater 1 and rater 2, respectively. This observation supports previous studies showing that unchanged ventricular size does not rule out shunt dysfunction [27]. This circumstance is especially common in long-term shunt-dependent patients. Periventricular gliosis leading to stiff ventricular walls and reduced compliance was previously thought to prevent ventricular enlargement [28]. This notion was then questioned by conducting infusion studies in shunted patients and showed a normal pressure gradient between intraventricular pressure and intraparenchymal pressure [27, 29]. Recent studies have reported that chronic cerebrospinal fluid (CSF) overdrainage modifies the dynamics and structure of the cerebral venous system, leading to pathological venous overdrainage. Long-term shunt-dependent patients suffering from chronic overdrainage show increased venous distensibility owing to low ICP as a result of CSF diversion, thus causing early collapse of cortical cerebral bridging veins when shunt dysfunction alters the transmural pressure gradient [30–32]. Therefore, shunted patients with chronic CSF overdrainage present with severe symptoms at an early stage before the ventricular system enlarges. A negative MR report and clinician’s uncertainty could lead to longer clinical observation and might cause delayed adequate treatment.

According to our knowledge, no imaging studies of shunt dysfunction have ever described MR venography as an additional imaging parameter that increases diagnostic certainty and decreases the number of false negative reports. As ventricular shunts are especially implanted in children with distorted ventricular anatomy, ventricular width might be evaluated as pathologic and can therefore not be used as a certain marker for shunt dysfunction without preliminary examination for comparison. Additionally, acquiring 3D vPCA in shunted children might therefore also increase diagnostic certainty if no preliminary examination is available.

During acquisition of 3D vPCA children should not move for 5–8 min or they may need to be sedated to achieve good image quality. Compared to the invasiveness of operative shunt revision with the patient under general anesthesia, a short sedation represents an acceptable intervention.

In this study raters were blinded for clinical symptoms of the patients. This is usually not the case in clinical routine, but incomplete clinical information may be a problem in teleradiology, where detailed clinical information cannot be obtained.

While so far it was known that according to image evaluation of children with ventriculoperitoneal shunt in cases of SF there are two groups, namely those with ventricular enlargement and those without [9, 17], by additionally evaluating venous sinuses using 3D vPCA, we were able to identify three groups:

1. widening of ventricles without compression of venous sinuses (Fig. 1a–d),
2. widening of ventricles with compression of venous sinuses (Fig. 1e–h),
3. compression of venous sinuses without changes in ventricular width (Fig. 1i–l).

Knowledge of these groups might raise the awareness that unchanged or slightly changed ventricular size does not rule out shunt dysfunction in children with clinical symptoms. Communicating the affiliation, especially to group 3 with the patient and/or their parents might help diagnostic work-up when admitted to a hospital where the patient is not known. The distribution among the three groups with the most children assigned to group 2 shows that in most cases the whole intracranial system seems to be affected; however, it also shows that there are cases where one system, either ventricles or veins, is exclusively affected and the reason remains unclear. To evaluate whether certain causes for ventricular shunting allow assignment to one of the three groups during the course, a larger patient cohort is needed.

The study has some limitations. Owing to its retrospective design MR examinations were performed on different MRI systems. Due to this fact and the long retrospective period with MR protocol changes, sequences were performed with slightly different parameters. This does not influence evaluation of ventricular width, because the contrast between CSF-filled spaces and brain tissue is not very sensitive to changes in TR and TE and therefore delineation of the ventricles is not affected. Spatial resolution differs in maximum about 0.23 mm^2^ in plane and 1 mm in slice thickness. Only one case was at one time point examined with a slice thickness of 4 mm. This resolution is sufficient to delineate the ventricles and evaluate for ventricular width.

The 3D vPCA also differed in spatial resolution between 0.43–0.9 mm^2^ and a slice thickness between 0.6 and 1.6 mm. Only one examination was performed with a slice thickness of 3 mm, but therefore the highest in-plane resolution. The most important factor for comparable phase contrast angiography is the velocity encoding, because this parameter influences visibility of vessels. This parameter is chosen depending in the maximum velocity expected in the system that is to be examined. Therefore, velocity encoding should be identical at least within follow up examinations. In this study there was one examination which was performed with a velocity encoding of 20 cm/s which is only 5 cm/s higher than all other examinations, so that this should not affect image evaluation as far as can be compared with literature [33–35], as velocity studies that can be found are made in adults or neonates [36]. The retrospective character of this study also leads to different time intervals between the two MR examinations of one patient reaching from 4 days to 3.5 years. As mentioned above influence on image impression by fusion of the fontanelles was prevented by excluding patients with long time intervals between both examinations younger than 4 years of age.

Furthermore, this procedure of evaluating ventricular width and sinus compression could be limited in patients younger than 1 year of age, who do not show a linear increase in ICP because the skull is more distensible due to open fontanelles and cranial sutures [37]. Additionally, if there is a large time gap between MR examinations that could be compared within the first 2 years of life, changes of ICP could lead to different image impressions because the fusion of fontanelles influences the morphologic consequences of elevated ICP. In our study we included 1 patient at the age of 1.5 years. MRI examinations were performed within 3 days, so that closure of the cranial sutures and fusion of the fontanelles could not influence image impression.

3D vPCA is an adequate additional MR marker of ICP and in cases of significant sinus compression without changes in ventricular width, as shown in Fig. 1i–l, could comprise a helpful tool in diagnosing SF; however, without baseline imaging under normal pressure conditions it holds the risk of producing false positive or false negative diagnoses due to sinus hypoplasia or anatomical variants caused by (intra)cranial malformations. Therefore, previous MRI studies render 3D vPCA more reliable.

The evaluated cohort of 20 children with ventriculoperitoneal shunt in whom 13 had a shunt dysfunction is quite small. Nevertheless, we could show that additional acquisition and evaluation of 3D vPCA in children with suspected SF improves diagnostic sensitivity, especially when presenting with unchanged ventricular width.

## Summary and Conclusion

This study showed that sinus compression is a reliable additional marker for elevated intracranial pressure (ICP) in this cohort of shunted pediatric patients, especially in those with constant ventricular width. With 10% of patients not showing elevated ICP due to shunt dysfunction by the widening of ventricles, the number is too high to neglect this group of patients in the MR protocol set-up for suspected shunt failure (SF). In our study this percentage is even higher with 31%. Therefore, additionally performing 3D venous phase contrast MR angiography (vPCA) in children with SF will lead to higher confidence in the diagnosis of SF for the reporting (neuro)radiologist and to a higher confidence in treatment initiation for the attending physician, leading to a faster adequate treatment of those patients. In order to confirm the indications for revision surgery in clinically unclear situations, 3D vPCA is a helpful additional tool. According to the results of our study, we recommend performing 3D vPCA in cases in which clinical symptoms suspect SF to accurately diagnose patients with intracranial hypertension, regardless of simultaneous enlargement of the ventricles or ventricular size remaining stable.

## Abbreviations

CSF: Cerebrospinal fluid
ICP: Intracranial pressure
LTS/RTS: Left/right transverse sinus
SF: Shunt failure
SSS: Superior sagittal sinus
vPCA: Venous phase-contrast angiography
